# Nobiletin Attenuates Inflammation and Modulates Lipid Metabolism in an In Vitro Model of Intestinal Failure-Associated Liver Disease

**DOI:** 10.3390/pharmaceutics18010087

**Published:** 2026-01-09

**Authors:** Marta Belka, Aleksandra Gostyńska-Stawna, Karina Sommerfeld-Klatta, Maciej Stawny, Violetta Krajka-Kuźniak

**Affiliations:** 1 Poznan University of Medical Sciences, Doctoral School, Bukowska 70, 60-812 Poznan, Poland; mbelka@ump.edu.pl; 2 Poznan University of Medical Sciences, Department Pharmaceutical Biochemistry, Rokietnicka 3, 60-806 Poznan, Poland; 3 Poznan University of Medical Sciences, Department of Pharmaceutical Chemistry, Rokietnicka 3, 60-806 Poznan, Poland; agostynska@ump.edu.pl (A.G.-S.); mstawny@ump.edu.pl (M.S.); 4 Poznan University of Medical Sciences, Department of Toxicology, Rokietnicka 3, 60-806 Poznan, Poland; ksommerfeld@ump.edu.pl

**Keywords:** IFALD, nobiletin, Nrf2, oxidative stress, NF-κB, hepatocytes

## Abstract

**Background**: Intestinal failure-associated liver disease (IFALD) is a serious complication in patients receiving parenteral nutrition, often exacerbated by inflammation, lipid overload, and oxidative stress. Nobiletin (NOB), a polymethoxylated flavone, is known for its anti-inflammatory and lipid-regulating properties. **Methods**: We employed an in vitro model using THLE-2 human hepatocytes and primary human cholangiocytes exposed to Intralipid (INT) and lipopolysaccharide (LPS) to simulate IFALD conditions. NOB was tested at non-toxic concentrations (10 and 25 µM) to assess its protective effects. MTT viability assays, multiplex bead-based immunoassays (MAGPIX), RT-qPCR, and Western blotting were used to evaluate changes in inflammation markers, gene expression, and protein signaling. Moreover, ALT and AST activities were used to assess hepatocellular injury. **Results**: NOB maintained high cell viability in THLE-2 hepatocytes and cholangiocytes, confirming its low cytotoxicity. NOB normalized ALT and AST activities in both tested cell lines, but the effect reached statistical significance only for ALT in cholangiocytes. Under IFALD-like conditions (LPS+INT), NOB significantly preserved metabolic activity in both cell types. In THLE-2 and cholangiocytes, NOB markedly reduced the phosphorylation of pro-inflammatory proteins JNK, NF-κB, and STAT3, indicating a broad inhibition of inflammatory signaling. Moreover, in THLE-2 cells, NOB upregulated lipid metabolism-related genes (*PRKAA2*, *CYP7A1*, and *ABCA1*) and decreased oxidative stress, thereby enhancing the nuclear translocation of Nrf2 and increasing SOD1 level, which supports the activation of antioxidant defenses. **Conclusions**: NOB exhibits hepatoprotective properties under IFALD-like conditions in vitro, likely through modulation of inflammation-related signaling and lipid metabolism pathways.

## 1. Introduction

Intestinal failure-associated liver disease (IFALD) is a serious complication that arises in patients with intestinal failure, particularly in neonates and children, who are dependent on long-term parenteral nutrition (PN). Clinically, IFALD manifests with cholestasis, hepatic steatosis, fibrosis, and progressive deterioration of liver function. The etiology of this disorder is multifactorial and complex, involving the interplay of inflammation, oxidative stress, and disturbances in bile acid and lipid metabolism [1]. Despite significant advances in supportive and nutritional care, effective pharmacological interventions for preventing and treating IFALD remain limited [2]. Therefore, a growing demand exists for novel therapeutic strategies to attenuate the liver injury associated with PN and restore hepatocellular homeostasis.

In recent years, bioactive natural compounds have received increasing attention as potential therapeutic agents for liver diseases. Among these, nobiletin (NOB), a polymethoxylated flavone predominantly present in the peel of citrus fruits, has been recognized for its broad spectrum of biological activities. Numerous experimental studies have demonstrated that NOB exhibits antioxidant, anti-inflammatory, lipid-regulating, and hepatoprotective properties [3]. The compound has been shown to modulate multiple intracellular signaling pathways, regulate gene expression patterns, and enhance the cellular defense response to oxidative stress. These pleiotropic effects suggest that NOB may be an attractive candidate for therapeutic application in liver conditions where inflammation and metabolic dysregulation play a central role, such as IFALD.

The protective effects of NOB are mediated by its ability to regulate several key molecular pathways. One of the most important is the Nrf2 (nuclear factor erythroid 2-related factor 2) pathway, a central regulator of the cellular defense against oxidative stress. Upon activation, Nrf2 translocates to the nucleus and induces the expression of detoxifying and antioxidant enzymes, including superoxide dismutase (SOD), heme oxygenase-1 (HO-1), and NAD(P)H quinone oxidoreductase 1 (NQO1) [4,5].

Oxidative stress is considered a key pathogenic factor in IFALD, as excessive production of reactive oxygen species (ROS) leads to mitochondrial dysfunction, lipid peroxidation, and hepatocellular injury. The Nrf2 pathway serves as a major regulator of antioxidant defense mechanisms, activating the expression of enzymes including SOD1, HO-1, and NQO1 [6]. Dysregulation of this pathway has been implicated in IFALD progression, where impaired Nrf2 activation contributes to oxidative damage and inflammatory amplification [7]. Therefore, the potential of NOB to modulate oxidative stress via the Nrf2 pathway represents an important therapeutic target in maintaining redox homeostasis and limiting liver injury.

On the other hand, NF-κB (nuclear factor kappa B) and STAT (signal transducer and activator of transcription) proteins, such as STAT3, are major mediators of inflammation, cytokine production, and stress responses. Dysregulated activation of NF-κB and STAT3 signaling cascades promotes persistent inflammation and contributes to hepatocellular damage in IFALD. In parallel, kinases such as JNK and ERK are known to control cell survival, apoptosis, and stress adaptation; their overactivation under inflammatory and lipid overload conditions further accelerates liver injury.

Metabolic imbalance is another important component of IFALD pathology. AMP-activated protein kinase (AMPK), which is encoded by the *PRKAA2* gene, acts as a cellular energy sensor, regulating lipid metabolism. *CYP7A1* encodes the rate-limiting enzyme in bile acid synthesis, while *ABCA1* plays a critical role in cholesterol efflux and reverse cholesterol transport. Dysregulation of these genes and associated pathways is strongly linked to hepatocyte steatosis, cholestasis, and lipid accumulation [8]. Therefore, targeting these signaling and metabolic pathways provides a rational strategy for preventing or mitigating the progression of IFALD.

To explore the hepatoprotective potential of NOB, we established an in vitro model of IFALD using immortalized human hepatocytes (THLE-2) and human cholangiocytes exposed to lipopolysaccharide (LPS) and Intralipid (INT), a combination that mimics the inflammatory and lipid overload conditions characteristic of PN-induced liver injury. This model allowed us to investigate how NOB influences inflammatory signaling cascades, lipid metabolism-related gene expression, and antioxidant defense mechanisms in THLE-2 cells and cholangiocytes exposed to IFALD-like stress.

In this study, primary emphasis was placed on assessing hepatocellular injury by measuring ALT and AST activities, followed by the analysis of inflammatory signaling molecules (JNK, NF-κB, STAT3), the expression of genes critical for lipid metabolism (*PRKAA2*, *CYP7A1*, and *ABCA1*), and the nuclear translocation of Nrf2, a key marker of antioxidant pathway activation. By comprehensively evaluating these parameters, we aimed to clarify the molecular mechanisms underlying the hepatoprotective effects of NOB and to determine its potential as a therapeutic candidate for IFALD.

## 2. Materials and Methods

### 2.1. Chemistry

NOB, 2-(3,4-dimethoxyphenyl)-5,6,7,8-tetramethoxy-4H-chromen-4-one (CAS: 478-01-3; catalog number: PA-03-8339-P; purity ≥ 98%) was purchased from Pol-Aura (Olsztyn, Poland), INT was purchased from Fresenius Kabi AB (Uppsala, Sweden), and LPS [CAS 93572-42-0] from Sigma-Aldrich (Saint Louis, MO, USA).

### 2.2. Cells and Culture Conditions

Human immortalized hepatocytes, THLE-2 (ATCC CRL-2706) cells, were provided by the American Type Culture Collection (ATCC, Manassas, VA, USA). THLE-2 cells were cultured in BEGM supplemented with the Bullet Kit (Lonza, Cologne, Germany) and 10% FBS, 5 ng/mL EGF, and 70 ng/mL phosphoethanolamine at 37 °C, in a humidified 5% CO_2_ atmosphere.

Human cholangiocyte primary cell culture (Sku: 36755-12) cells were provided by Celprogen (Torrance, CA, USA). Cholangiocytes were cultured in human cholangiocyte primary cell culture complete media with serum (Celprogen, Torrance, CA, USA) at 37 °C in a humidified 5% CO_2_ atmosphere.

### 2.3. Viability Assay

The effect of NOB on cell viability was assessed by the MTT assay, following the standard protocol. Briefly, THLE-2 cells and cholangiocytes were seeded (10^4^ per well) in 96-well plates. After 24 h of preincubation, compounds were added at concentrations ranging from 1 to 100 µM, and the cells were incubated for an additional 24 h. Later, the cells were washed twice with phosphate-buffered saline (PBS) and further incubated for 4 h with a medium containing 0.5 mg/mL 3-(4,5-dimethylthiazol-2-yl)-2,5-diphenyltetrazolium bromide (MTT). Then, the formazan crystals were dissolved in acidic isopropanol, and the absorbance was measured at 570 nm and 690 nm. Cell viability was calculated relative to untreated control cells, and the assay was used to identify non-cytotoxic NOB concentrations for subsequent experiments; therefore, no dedicated positive cytotoxic control was included. Based on these results, NOB concentrations of 10 and 25 µM, corresponding to viability levels above 75%, were selected for all further analyses.

### 2.4. IFALD Model

The developed model mimics IFALD-like conditions by combining inflammatory stimulation (LPS) with lipid overload (INT). THLE-2 cells and cholangiocytes were first exposed to INT (10 mg/mL) for 1 h, after which LPS (0.1 µg/mL) was added, and the cells were incubated for another 24 h. To assess the effect of NOB, the compound was co-administered with LPS at concentrations of 10 or 25 µM, followed by a 24 h incubation. The untreated cells served as a negative control to establish a physiological baseline, while the group exposed to INT+LPS without NOB treatment was used as an internal reference for maximal cellular injury. Additionally, an INT-alone control was included to differentiate between the metabolic stress caused by lipid overload and the combined inflammatory-metabolic injury induced by the INT+LPS model.

### 2.5. Nuclear, Cytosolic, and Total Protein Lysate Preparation

The subcellular extracts from THLE-2 cells treated with INT, LPS and NOB were prepared using the Nuclear/Cytosol Fractionation Kit (BioVision Research, Milpitas, CA, USA. In summary, THLE-2 cells were obtained by centrifugation at 600× *g* for 5 min at 4 °C. Dithiothreitol (DTT) and protease inhibitors were added to an ice-cold cytosol extraction buffer before pellets were resuspended. The samples were centrifuged at 16,000× *g* for 5 min at 4 °C to extract the cytosolic fractions after being incubated on ice for 10 min. Clean tubes were filled with the supernatants—cytosolic fractions. After being resuspended in an ice-cold nuclear extraction solution containing protease inhibitors and DTT, the pellets were incubated on ice for 40 min, with vortexing every 10 min for 15 s. After centrifuging the lysed nuclei suspension at 16,000 g for 10 min at 4 °C, the nuclear extract was recovered and kept at −80 °C [9]. Lysates from THLE-2 cells and cholangiocytes were prepared using Radioimmunoprecipitation Assay (RIPA) buffer supplemented with protease inhibitors (Sigma-Aldrich, Saint Louis, MO, USA). The samples were stored at −80 °C for future downstream applications.

### 2.6. Enzymatic Detection of Aminotransferases

Alanine aminotransferase (ALT) and aspartate aminotransferase (AST) activity were quantitatively determined using in vitro enzymatic tests based on coupled enzymatic reactions measured with a Cobas pure c 303 automated chemistry analyzer (Roche Diagnostics, Mannheim, Germany). The commercial reagents used were the ALTP2 test for ALT and the ASTP2 test for AST (both from Roche Diagnostics). The aminotransferases (ALT/AST) catalyze the transfer of an amino group from an amino acid (L-alanine for ALT; L-aspartate for AST) to 2-oxoglutarate, resulting in the formation of L-glutamate and a specific keto acid (pyruvate or oxaloacetate). In a secondary “indicator” reaction, this keto acid reacts with NADH in the presence of a specific dehydrogenase (LDH for ALT; MDH for AST), forming a hydroxy acid and NAD+. Pyridoxal phosphate (PLP) serves as the essential coenzyme for the initial transamination, ensuring full enzyme activation. Because the consumption of NADH is measured at 340 nm, the rate of NADH oxidation is directly proportional to the catalytic activity of the aminotransferase. The AST/ALT measurement specifically employs pyridoxal phosphate activation and adheres to the recommendations set forth by the International Federation of Clinical Chemistry (IFCC); however, the manufacturer has internally optimized the assay for operational performance and stability. The results are typically expressed in international units per liter (IU/L).

### 2.7. Bead-Based Immunoassay on the Luminex MAGPIX

A magnetic bead-based multiplex immunoassay was performed using the Luminex MAGPIX^®^ platform. A commercially available (not custom-made) high-sensitivity magnetic bead panel (MILLIPLEX^®^ MAP Multi-Pathway Total/Phosphoprotein Magnetic Bead Kit Merck/Millipore Sigma, Darmstadt, Germany) was used to quantify, in lysates of treated THLE-2 cells and cholangiocytes, JNK, phospho-JNK, NF-κB, phospho-NF-κB, STAT3, and phospho-STAT3, according to the manufacturer’s instructions. Briefly, cell lysates were diluted in MILLIPLEX^®^ MAP buffer and transferred to a 96-well plate. A suspension of antibody-coupled magnetic beads was added to each well, followed by samples, and the plate was incubated overnight at 2–8 °C on a shaker, protected from light. After washing, MILLIPLEX^®^ MAP detection antibodies were added and incubated for 1 h at room temperature with shaking. Subsequently, streptavidin–phycoerythrin (SAPE) was added to generate the reporter signal, and an additional signal-enhancement step was performed using MILLIPLEX^®^ MAP Amplification Buffer (15 min, room temperature). Beads were then resuspended in MILLIPLEX^®^ MAP buffer and acquired on the MAGPIX^®^ analyzer, which identifies each bead region and records reporter fluorescence. Raw mean fluorescence intensity (MFI) values were collected using Luminex XPONENT for MAGPIX software, version 4.2 (Luminex Corporation, Austin, TX, USA) and further processed in MILLIPLEX^®^ Analyst 5.1 (EMD Millipore, Burlington, MA, USA). For data presentation, MFI values were normalized to the untreated control (relative to control).

### 2.8. Total RNA Isolation, cDNA Synthesis, and Quantitative Real-Time PCR

Total RNA was isolated using the GeneMatrix Universal DNA/RNA/Protein Purification Kit (EURx, Gdansk, Poland). The obtained RNA samples were then reverse-transcribed into cDNA with the RevertAid First Strand cDNA Synthesis Kit (Thermo Fisher Scientific, Waltham, MA, USA) following the manufacturer’s protocol. For the quantitative Real-Time PCR analyses, the Maxima SYBR Green Kit (Fermentas Inc., Waltham, MA, USA) and the LightCycler 96 (Roche, Basel, Switzerland) were used. The protocol began with 5 min of enzyme activation at 95 °C, followed by 40 cycles of 95 °C for 15 s, 56 °C for 20 s, and 72 °C for 40 s, and concluded with a final elongation at 72 °C for 5 min. Amplicon specificity was confirmed by melting curve analysis. Gene expression levels were normalized to *TBP (TATA box-binding protein)* and *PBGD (porphobilinogen deaminase)* as reference genes. Relative changes in gene expression were calculated using the Pfaffl method.

The primer sequences, listed in Table 1, were designed using Beacon Designer software version 7.9 and subjected to a BLAST search to minimize nonspecific binding. They were then synthesized at the Institute of Biochemistry and Biophysics, Polish Academy of Sciences (Warszawa, Poland).

### 2.9. Western Blot Analysis

Cytosolic and nuclear fractions were resolved by SDS–PAGE using Mini-PROTEAN^®^ TGX Stain-Free™ gels (Bio-Rad Laboratories, Hercules, CA, USA) with 100 µg of protein loaded per lane, and proteins were transferred onto PVDF membranes (Immobilon^®^ P; Sigma-Aldrich, St. Louis, MO, USA). After electrophoresis, Stain-Free™ total protein images were acquired under UV activation using a ChemiDoc™ imaging system (Bio-Rad Laboratories, Hercules, CA, USA). Following transfer, membranes were also imaged to confirm protein transfer and to obtain lane-specific total protein signals. The Stain-Free lane total protein signal was used for total protein normalization (TPN) of target bands, as recommended for quantitative Western blotting and as an alternative to single housekeeping proteins [10]. Target proteins were detected using primary antibodies against Nrf2 and SOD1 (Santa Cruz Biotechnology, Dallas, TX, USA), followed by AP- or HRP-conjugated secondary antibodies (Santa Cruz Biotechnology, Dallas, TX, USA and BosterBio, Pleasanton, CA, USA). The protein–antibody complexes were visualized using AP Conjugate Substrate Kit NBT/BCIP and Chemiluminescent HRP substrate of Clarity™ Western ECL kit (Bio-Rad, Hercules, CA, USA). Densitometric quantification of bands was performed using Image Lab™ software, version 6.1.0 (Bio-Rad, Hercules, CA, USA).

### 2.10. Oxidative Stress Analysis

Intracellular ROS production was assessed using dihydroethidium (DHE), which selectively reacts with superoxide anions to form DNA-binding fluorescent products. Quantitative analysis of superoxide radicals was performed with the Muse^®^ Oxidative Stress Kit (Merck KGaA, Darmstadt, Germany) in accordance with the manufacturer’s instructions. After 24 h of incubation, cells were trypsinized, washed with PBS, and resuspended in Assay Buffer containing the Muse Oxidative Stress Reagent working solution. Fluorescence signals corresponding to ethidium bromide bound to cellular DNA in superoxide-positive cells were detected using the Muse^®^ Cell Analyzer (Merck KGaA, Darmstadt, Germany). The proportions of ROS-positive and ROS-negative cells were determined with Muse^®^ Software version 1.4 (Merck KGaA, Darmstadt, Germany) and compared with those of the negative control (untreated cells).

### 2.11. Statistical Analysis

Statistical analysis and graphs were calculated and prepared using GraphPad Prism (GraphPad Software, version 9, San Diego, CA, USA), with a significance level of *p* < 0.05. The Dunnett’s test was used to assess the statistical significance relative to the untreated control cells or to the INT+LPS-treated group.

## 3. Results

### 3.1. Nobiletin Preserves Cell Viability

**Figure 1:**
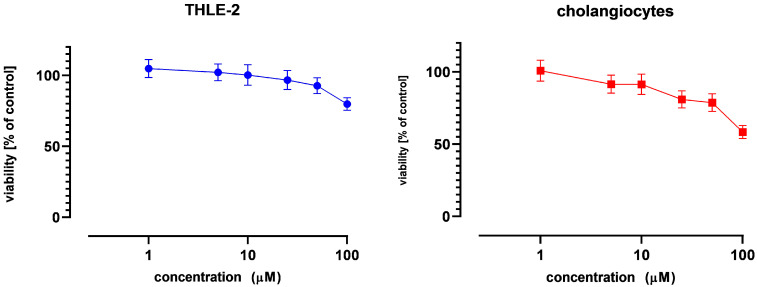
The effect of nobiletin (NOB) on the viability of THLE-2 and cholangiocyte cells after 24 h incubation. Data (mean ± SEM) from three independent experiments run in duplicate are presented.

The effect of NOB on the viability of THLE-2 hepatocytes and primary human cholangiocytes was evaluated using the MTT assay. NOB exhibited negligible cytotoxicity in both cell types, maintaining viability above 75% within the tested concentration range of 1–100 µM (Figure 1). The calculated IC_50_ value exceeded 100 µM, indicating high cellular tolerance and favorable safety characteristics. Based on these results, concentrations of 10 µM and 25 µM were selected for subsequent in vitro experiments. The selection of the stress factors LPS (0.1 µg/mL) [11,12] and INT (10 mg/mL) was based on previously published protocols [13]. Overall, our observations are consistent with prior reports indicating that NOB exhibits minimal cytotoxicity in hepatocyte models and primary hepatic cells [14], thereby supporting its potential therapeutic application in liver-related disorders.

### 3.2. Nobiletin Lowers ALT and AST Liver Enzyme Activity

**Figure 2:**
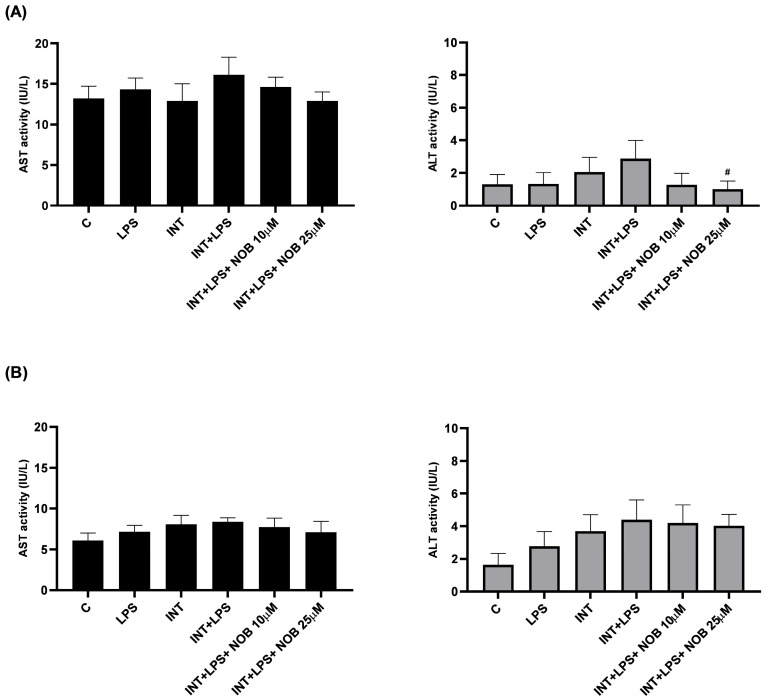
Effect of NOB, LPS, and INT on the AST and ALT activity in THLE-2 (**A**) and cholangiocytes (**B**) cells. Data represent mean ± SEM from two independent experiments run in triplicate. Statistical significance was assessed using Dunnett’s test. The hashtag above denotes statistical significance relative to the treated INT+LPS-treated cells with # *p* < 0.05.

To assess hepatocellular injury under IFALD-like conditions, the activities of alanine aminotransferase (ALT) and aspartate aminotransferase (AST) were determined in cell supernatants (Figure 2). In THLE-2 hepatocytes, exposure to INT+LPS resulted in increased activity of AST and ALT, but not significantly compared to the untreated control cells. The addition of NOB at concentrations of 10 and 25 µM partially restored the activity of both enzymes toward control levels, with the 25 µM dose producing a statistically significant reduction of ALT in THLE-2 cells compared with the INT+LPS group. No significant effects of NOB were observed in cholangiocytes for either enzyme.

### 3.3. Nobiletin Suppresses Inflammatory Kinase Activation and Signaling Pathways

**Figure 3:**
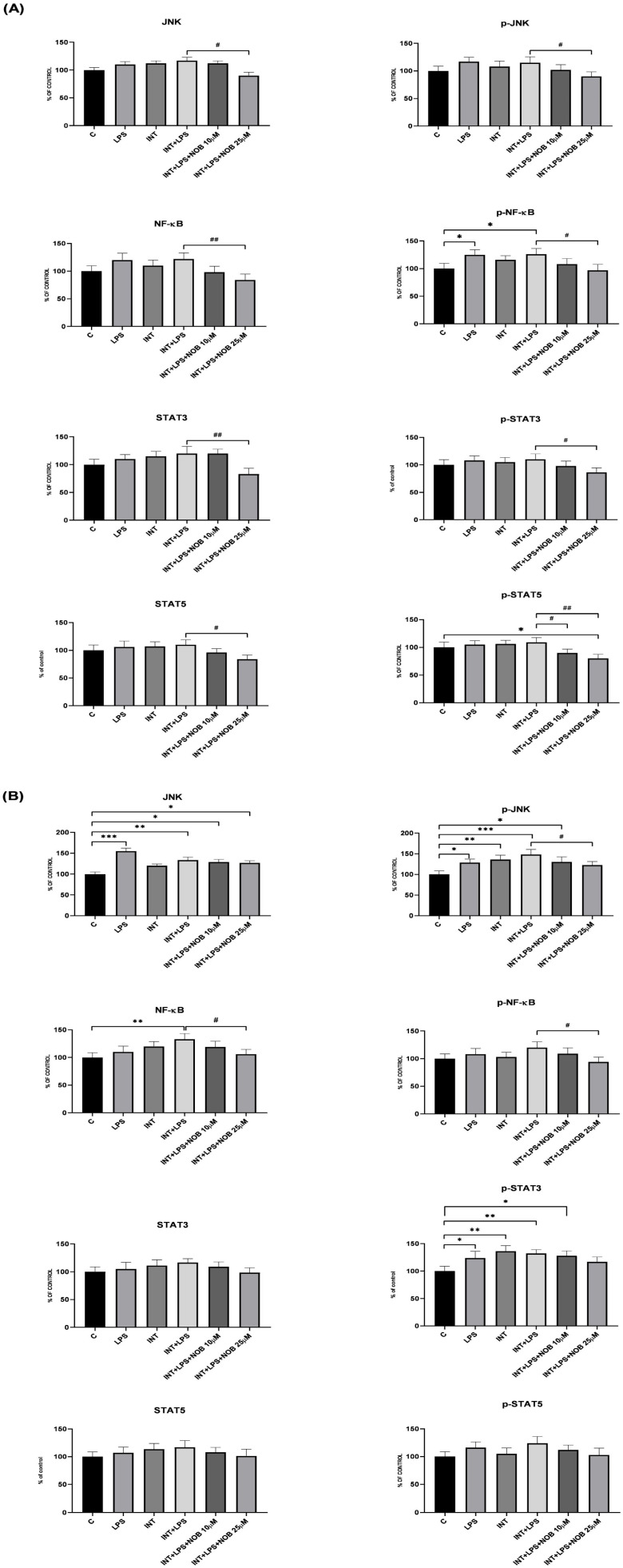
Effect of NOB, LPS, and INT on the JNK, p-JNK, NF-κB, p-NF-κB, STAT3, p-STAT3, in THLE-2 (**A**) and cholangiocytes (**B**) cells. Total protein and phosphorylated levels were quantified using fluorescence intensity (MFI) values obtained with the MAGPIX^®^ system. Data represent mean ± SEM from two independent experiments run in triplicate, and are expressed as fold change relative to untreated control cells (set to 100%). Statistical significance was assessed using Dunnett’s test. Asterisks above denotes statistical significance relative to the untreated control cells with * *p* < 0.05, ** *p* < 0.01, *** *p* < 0.001. Hashtags above denotes statistical significance relative to the INT+LPS-treated cells with # *p* < 0.05, ## *p* < 0.01.

In our IFALD model, exposure to LPS and INT markedly enhanced the phosphorylation of several kinases and transcription factors involved in inflammatory signaling, including JNK, NF-κB, and STAT3. Treatment with NOB significantly reversed these effects. Specifically, in THLE-2 hepatocytes, 25 µM NOB reduced NF-κB phosphorylation by approximately 30% compared with LPS+INT-treated cells. Similarly, phosphorylation of JNK and STAT3 decreased by about 25% and 22%, respectively (Figure 3A). In cholangiocytes, exposure to LPS and INT also markedly enhanced the phosphorylation of JNK, NF-κB, and STAT3, confirming robust activation of pro-inflammatory signaling cascades under IFALD-like conditions. NOB treatment substantially attenuated these effects in a concentration-dependent manner. At 25 µM, phosphorylation levels of NF-κB and JNK were reduced by approximately 20–25%, while STAT3 phosphorylation did not decrease significantly compared with the LPS+INT group (Figure 3B). Notably, the total protein levels of the analyzed signaling molecules remained unchanged, indicating that NOB primarily modulates the phosphorylation status rather than protein expression. These findings demonstrate that NOB effectively suppresses inflammatory signaling activation not only in hepatocytes but also in biliary epithelial cells, suggesting its potential role in preventing cholangiocyte-mediated inflammatory and cholestatic injury in IFALD.

### 3.4. Nobiletin Modulates the Expression of Lipid Metabolism-Related Genes

**Figure 4:**
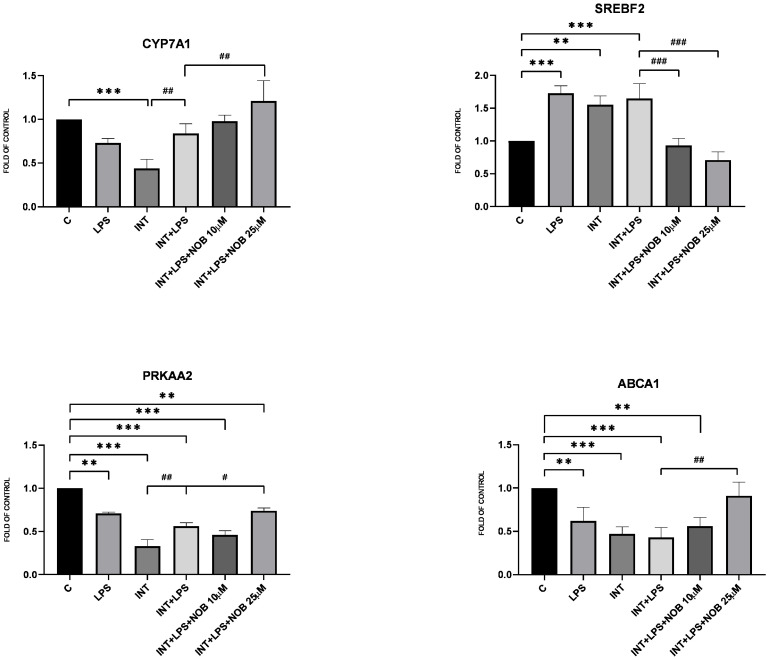
Effect of NOB, LPS, and INT on *PRKAA2*, *SREBF2*, *CYP7A1*, and *ABCA1* mRNA levels after 24 h incubation. Data (mean ± SEM) represent relative mRNA expression in THLE-2 cells from two independent experiments run in triplicate, and are expressed as fold change relative to untreated control cells (set to 1). Statistical significance was assessed using Dunnett’s test. Asterisks above denotes statistical significance relative to the untreated control cells with ** *p* < 0.01, *** *p* < 0.001. Hashtags above denotes statistical significance relative to the INT+LPS-treated cells with # *p* < 0.05, ## *p* < 0.01, ### *p* < 0.001.

Disruption of lipid metabolism is a hallmark of IFALD pathogenesis, characterized by steatosis, impaired bile acid homeostasis, and altered cholesterol efflux. In our study, NOB modulated the expression of key lipid metabolism-related genes in hepatocytes exposed to INT+LPS. Treatment with NOB at 25 µM significantly increased the expression of *PRKAA2* (by approximately 40%), *CYP7A1* (by about 50%), and *ABCA1* (by nearly 45%) compared with the INT+LPS group (Figure 4, and Table S1). Additionally, the expression of *SREBF2*, a key transcription factor involved in cholesterol biosynthesis, was evaluated. Exposure to INT+LPS increased *SREBF2* mRNA levels compared with control cells. Whereas treatment with NOB markedly reduced the expression of *SREBF2*, resulting in an approximately 60% decrease relative to INT+LPS-treated cells.

### 3.5. Nobiletin Attenuates Oxidative Stress Through Nrf2 Activation

**Figure 5:**
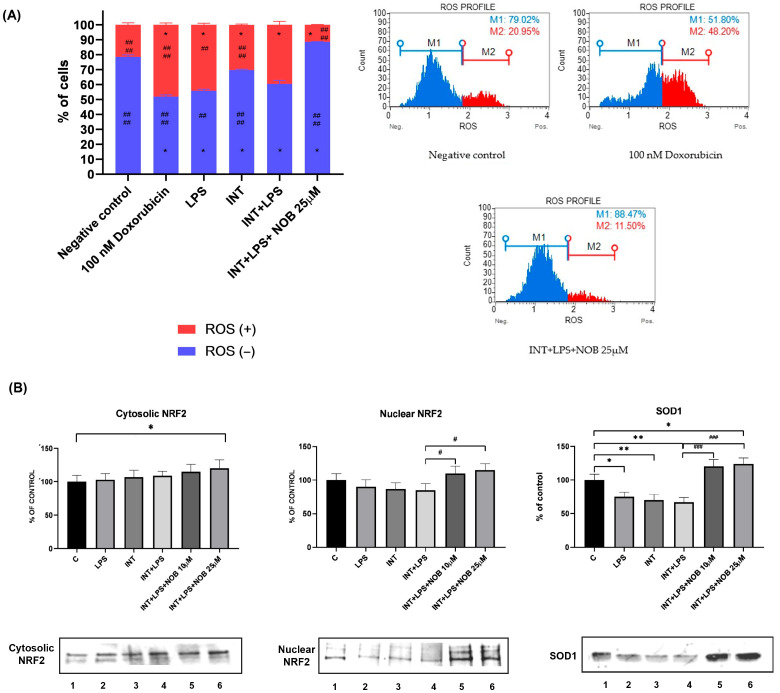
Effect of NOB, LPS, and INT on oxidative stress (**A**) and Nrf2 signaling pathways (**B**). ROS levels in THLE-2 cells after 24 h treatment with LPS, INT, INT+LPS, and INT+LPS+NOB (25 µM). Non-treated cells and DOXO (100 nM) served as negative and positive controls, respectively. ROS (+) and ROS (−) indicate cells with detectable or undetectable superoxide radicals, respectively. Representative histograms are shown, and data represent mean ± SEM from two independent experiments run in duplicate. Nrf2 subcellular distribution (cytosolic and nuclear fractions) and cytosolic SOD1 levels in THLE-2 cells after 24 h treatment. Representative blots are shown along with the corresponding Stain-Free™ total protein images used for total protein normalization (TPN). Lane order: (1) control, (2) LPS, (3) INT, (4) INT+LPS, (5) INT+LPS+NOB (10 µM), (6) INT+LPS+NOB (25 µM). Band intensities were quantified by densitometry and normalized to the lane-specific total protein signal obtained from Stain-Free imaging. Data are presented as mean ± SEM from two independent experiments, each performed in duplicate. Asterisks above denotes statistical significance relative to the untreated control cells with * *p* < 0.05, ** *p* < 0.01. Hashtags above denotes statistical significance relative to the INT+LPS-treated cells with # *p* < 0.05, ## *p* < 0.01, ### *p* < 0.001.

Oxidative stress represents a key pathogenic mechanism contributing to hepatocellular injury under IFALD-like conditions. To determine whether NOB modulates the redox status of hepatocytes, intracellular oxidative stress levels were assessed in THLE-2 cells exposed to LPS and INT. Exposure to LPS or the combination of LPS and INT significantly increased oxidative stress compared with control cells, confirming the activation of pro-oxidant pathways and impairment of the cellular redox balance. Treatment with NOB significantly attenuated oxidative stress and restored redox homeostasis (Figure 5A). To further substantiate this mechanism, the distribution of Nrf2 between cytosolic and nuclear fractions was analyzed in THLE-2 hepatocytes, which accurately reflect hepatic oxidative stress responses in the IFALD model. Co-treatment with NOB (10 and 25 µM) markedly enhanced nuclear Nrf2 accumulation, with the 25 µM dose producing ~25% higher levels than LPS+INT-treated cells (Figure 5B and Figure S1). To further investigate the antioxidant effects of NOB, we examined the SOD1 level, a key enzyme involved in detoxifying superoxide radicals. LPS+INT exposure resulted in a ~40% reduction in SOD1 protein levels compared to untreated cells, indicating impaired antioxidant defense. Remarkably, NOB treatment significantly restored SOD1 level, with the most substantial effect observed at 25 µM, which approached higher levels of control cells. Our findings are consistent with earlier studies [15], which report that NOB increases Nrf2 activation, induces downstream antioxidant enzymes such as HO-1 and NQO1, and reduces oxidative liver injury in diet- or toxin-induced models.

## 4. Discussion

In this study, an in vitro model of IFALD was developed and applied, based on human immortalized hepatocytes (THLE-2) and human cholangiocytes. Both cell types were analyzed independently in separate experiments, allowing for the reproduction of two key aspects of IFALD pathophysiology: hepatocyte injury induced by inflammation and lipid overload, and cholangiocyte dysfunction leading to cholestasis. This dual approach captured both the inflammatory-metabolic and cholestatic components that together define the clinical manifestations of IFALD.

An important feature of this model is the implementation of the THLE-2 cell line (ATCC CRL-2706), which is derived from normal human hepatocytes immortalized with the SV40 large T antigen. These cells retain most of the morphological and functional features of liver cells, including the expression of key enzymes involved in lipid metabolism, transport proteins, and signaling pathways responsive to oxidative and inflammatory stress [16]. The model employs INT, a soybean oil-based lipid emulsion rich in phytosterols, and LPS, a bacterial endotoxin that elicits a potent inflammatory response. The combination of these two factors enables the simulation of the main pathogenic mechanisms of IFALD: lipid overload and chronic inflammation.

From a methodological standpoint, the developed model offers several important advantages. First, the use of human, non-neoplastic cell lines allows for a more accurate reflection of hepatic physiology compared to commonly used hepatoma lines such as HepG2. The application of INT as a source of lipid stress enables modeling of phytosterol effects, which, through antagonism of the FXR receptor, play a crucial role in the development of cholestasis and bile acid metabolism disorders in IFALD. The combination of INT and LPS produces a “double-hit” effect, in which metabolic and inflammatory factors synergistically intensify cellular stress [17]. Moreover, the inclusion of cholangiocytes in separate experiments represents a significant methodological advancement, as biliary epithelial cells are particularly susceptible to the toxic effects of phytosterols and FXR dysregulation. This enables the investigation of the cholestatic component of IFALD, which cannot be captured in models that are exclusively hepatocyte-based.

Several in vitro models of IFALD have been described in the literature [11,12,18,19], differing in cell type, mechanisms represented, and translational relevance (Table 2). The THLE-3 model, derived from a similar origin, has been used to study epithelial–mesenchymal transition (EMT) and fibrosis induced by various parenteral nutrition emulsions (Omegaven, Smoflipid, Clinoleic). Unlike THLE-2, it focuses primarily on structural remodeling and fibrogenesis rather than inflammatory responses. Despite its widespread use, the HepG2 cell line exhibits a neoplastic phenotype and altered lipid metabolism, which limits its translational applicability.

Animal-derived models, such as BRL rat hepatocytes with *IRE1α* knockout or primary rabbit hepatocytes, have provided insights into endoplasmic reticulum stress and lipid accumulation. However, interspecies differences in bile acid metabolism and FXR regulation restrict their relevance. Models based on primary human monocytes, used to assess the effects of parenteral nutrition emulsions on cytokine secretion, are complementary but do not reproduce the metabolic processes typical of hepatocytes.

In this context, THLE-2 hepatocytes and primary cholangiocytes offer an advantageous in vitro platform that balances physiological relevance with experimental control. These models enable exploration of key aspects of IFALD pathogenesis, including inflammation, oxidative stress, and cholestasis. Although they lack immune and endothelial components, they provide a robust foundation for mechanistic studies and serve as a preparatory step toward more advanced co-culture systems or three-dimensional microfluidic platforms.

This study demonstrated that NOB exerts a pronounced hepatoprotective effect in an in vitro model of THLE-2 hepatocytes and cholangiocytes exposed to inflammatory and lipid stress, simulating IFALD-like conditions. Our findings indicate that NOB can modulate inflammatory pathways and regulate lipid metabolism, supporting its therapeutic potential in managing liver complications associated with parenteral nutrition.

The analysis of ALT and AST in THLE-2 hepatocytes and cholangiocytes treated with stress factors revealed that the combined exposure to INT and LPS induced changes in liver cells characteristic of inflammatory and lipotoxic injury (Figure 2). Following treatment with NOB, the activity of both enzymes decreased to levels close to or lower than those observed in the controls, suggesting an attenuation of metabolic stress and the restoration of cellular homeostasis. This effect is consistent with previously described actions of polyphenols, which alleviate oxidative stress, stabilize mitochondria, and improve the metabolic functions of liver cells [20]. The higher variability in THLE-2 observed for AST compared to ALT in our study likely reflects both biological and methodological differences between these enzymes. ALT is a more hepatocyte-specific and cytosolic marker, whereas AST is present in both cytosolic and mitochondrial compartments and may be less sensitive to mild or early cellular injury, particularly in acute in vitro models [21]. Given the relatively short exposure time (24 h) and the absence of extensive cell death, AST changes may remain subtle and fall below the threshold of statistical significance despite showing a consistent trend. In contrast, ALT, which is considered a more sensitive indicator of hepatocellular injury under these conditions, showed a significant reduction following NOB treatment.

Consistent with its known metabolic effect, NOB modulated the expression of genes involved in lipid metabolism. In THLE-2 hepatocytes, NOB increased the expression of *PRKAA2*, *CYP7A1*, and *ABCA1*, indicating activation of pathways promoting lipid catabolism and bile acid synthesis. Among these genes, a significant effect was observed for *CYP7A1*, which encodes the rate-limiting enzyme of bile acid synthesis. This observation is particularly relevant given that disturbances in bile acid metabolism represent an important pathogenic feature of IFALD. Consistent with these findings, previous work in a non-alcoholic fatty liver disease (NAFLD) model has shown that NOB reduces hepatic triglyceride and cholesterol accumulation through AMPK activation and suppression of lipogenic pathways such as SREBP-1c and FAS [15]. In addition, NOB has been reported to improve bile acid metabolism via induction of *CYP7A1* expression in high-fat diet-fed mice, further supporting its role in regulating lipid handling under conditions of metabolic overload [4]. Collectively, these data support the view that NOB contributes to the restoration of lipid metabolic balance in hepatocytes exposed to lipid overload under IFALD-like conditions.

In addition to its anti-inflammatory and metabolic effects, NOB activated the Nrf2 pathway, a central defense mechanism against oxidative stress. In THLE-2 cells, NOB markedly increased nuclear accumulation of Nrf2 and restored SOD1 protein expression suppressed by LPS+INT exposure. These findings indicate that NOB enhances antioxidant defenses and reinforces cellular redox homeostasis, consistent with previous reports showing its ability to suppress oxidative stress in various models of liver injury, including high-fat diet- and toxin-induced hepatotoxicity [4,5,15]. In particular, Fan et al. demonstrated that NOB enhanced the Nrf2–HO-1–NQO1 signaling axis, attenuated oxidative damage, and improved hepatocellular redox homeostasis in a high-fat diet-induced model of liver injury [15]. Consistently, Ke et al. reported that NOB supplementation increased hepatic Nrf2 activation and decreased malondialdehyde (MDA) levels, indicating a reduction in lipid peroxidation [4]. Excessive accumulation of ROS disrupts mitochondrial function, promotes lipid peroxidation, and amplifies inflammatory signaling, thereby exacerbating hepatocellular injury. Therefore, the ability of NOB to activate the Nrf2 pathway is particularly relevant in IFALD, a disease in which oxidative stress plays an important role in both the onset and progression. Through concurrent activation of Nrf2-dependent antioxidant defenses and suppression of inflammatory parameters (JNK, NF-κB, STAT3), NOB may help maintain redox homeostasis and limit secondary inflammatory amplification. Restoration of Nrf2 activity may further protect against mitochondrial dysfunction, thereby contributing to the attenuation of IFALD-related liver injury. Nevertheless, it should be highlighted that although NOB-induced Nrf2 activation coincided with suppression of NF-κB, JNK, and STAT3 signaling, the present study does not establish a direct causal link between these pathways. While extensive crosstalk between Nrf2 and inflammatory pathways has been reported in the literature [22,23], confirming whether NOB-mediated inhibition of NF-κB, STAT3, or JNK is Nrf2-dependent would require targeted loss-of-function approaches.

A notable strength of this study is the use of an in vitro IFALD model that allows mechanistic interrogation of inflammatory signaling, lipid overload-driven metabolic responses, and redox regulation in hepatocytes and cholangiocytes under IFALD-like conditions. Nevertheless, the study also has limitations. By design, this reductionist in vitro approach focuses on hepatocyte- and cholangiocyte-intrinsic responses to acute lipid overload and endotoxin exposure. Consequently, the absence of immune and stromal components limits the analysis primarily to early inflammatory and functional alterations and does not fully capture tissue-level remodeling or fibrogenesis. In addition, cholangiocyte analyses were restricted to general cytoprotective and inflammatory responses and did not include cholangiocyte-specific functional readouts, such as CFTR- or AE2-mediated secretory activity, nor markers of biliary identity and differentiation (e.g., KRT19 and KRT7) or epithelial barrier integrity. Incorporation of these endpoints in future studies will be necessary to more comprehensively characterize cholangiocyte dysfunction and its contribution to the cholestatic features of IFALD. Accordingly, the current model should be regarded as a first-line screening and hypothesis-generating platform to be complemented by more complex multicellular systems in subsequent work.

## 5. Conclusions

In summary, NOB demonstrated potent hepatoprotective and cholangioprotective effects in an in vitro IFALD model. It preserved cell viability, reduced inflammation by lowering ALT and AST levels, and suppressed the phosphorylation of key pro-inflammatory proteins (NF-κB, JNK, STAT3). Moreover, NOB decreased the level of ROS and enhanced antioxidant defense through Nrf2 activation and upregulation of SOD1 in THLE-2 hepatocytes. In addition, NOB modulated the expression of genes related to lipid metabolism, providing mechanistic insight into its potential role in metabolic regulation, although further functional validation is required. These combined actions highlight NOB as a promising candidate for further preclinical evaluation in the prevention and treatment of IFALD.

## Figures and Tables

**Table 1:** The sequence of starters used in real-time PCR reactions.

Primer		Sequence	Product Size
*PBGD*	forward	5’CCGCATCTGGAGTTCAGGAGTATTC	101 bp
	reverse	5’CCAGCTGTTGCCAGGATGATG	
*TBP*	forward	5’GGCACCACTCCACTGTATC	183 bp
	reverse	5’GGGATTATATTCGGCGTTTCG	
*ABCA1*	forward	5’TGAGGGAACATGGCTTGTT	143 bp
	reverse	5’CTCAGCCGAACAGAGATCAG	
*SREBF2*	forward	5’AACGGTCATTCACCCAGGTC	133 bp
	reverse	5’GGCTGAAGAATAGGAGTTGCC	
*CYP7A1*	forward	5’CATTTGGGCACAGAAGCATTG	174 bp
	reverse	5’AGGCAGCGGTCTTTGAGTTAG	
*PRKAA2*	forward	5’TCAATCGTTCTGTCGCCAC	530 bp
	reverse	5’ATACGGTTTGCTCTGACTTCG	

**Table 2:** Methodological comparison of cell models used in IFALD research.

Cell Model	Type of Cells	Stress Factors	Advantages	Limitations	Reference
Human monocytes	Primary human monocytes isolated from peripheral blood	Omegaven, Smofipid, Clinoleic, Lipofundin + LPS	Reflects the immune and cytokine response to lipid emulsions	Lacks hepatocyte-specific metabolic pathways	[11]
THLE-3	Immortalized human liver epithelial cells	Omegaven, Clinoleic, Lipofundin, Smoflipid + TGF-β1	Useful for modeling early fibrotic responses; maintains epithelial features	Limited expression of CYP450 enzymes; weak inflammatory responsiveness	
Primary rabbit hepatocytes	Neonatal rabbit hepatocytes	Soybean oil-based lipid emulsion *vs*. fish oil-based lipid emulsion	Physiological hepatocyte phenotype; responsive to lipid overload	Short lifespan; limited stability; species-specific differences in bile acid metabolism and FXR signaling	[18]
HepG2	Human hepatocellular carcinoma	Omegaven +LPS	Easy to culture; widely available	Neoplastic phenotype; limited metabolic fidelity	[12]
		Intralipid, EPA and DHA, Omegaven + TNFα, TGF-β,			[19]

## Data Availability

The original contributions presented in this study are included in the article/Supplementary Material. Further inquiries can be directed to the corresponding author.

## Supplementary Materials

The following supporting information can be downloaded at: https://www.mdpi.com/article/10.3390/pharmaceutics18010087/s1, Table S1. Raw PCR data for gene expression analysis: (a) the levels of TBP, PGGD; (b) the levels of CYP7A1, SREPF2; (c) the levels of PRKAA2, ABCA1. Figure S1. The representative Western blot images of cytosolic and nuclear Nrf2, and cytosolic SOD1.

## Abbreviations

The following abbreviations are used in this manuscript:

*ABCA1*	ATP-binding cassette transporter A1
AE2	Anion exchanger 2
ALT	Alanine aminotransferase
AMPK	AMP-activated protein kinase
AST	Aspartate aminotransferase
BEGM	Bronchial epithelial cell growth medium
BRL	Buffalo rat liver
CFTR	Cystic fibrosis transmembrane conductance regulator
*CYP7A1*	Cholesterol 7 alpha-hydroxylase
CYP450	Cytochrome P450
EMT	Epithelial–mesenchymal transition
ERK	Extracellular signal-regulated kinase
FXR	Farnesoid X receptor
HO-1	Heme oxygenase-1
IFALD	Intestinal failure-associated liver disease
INT	Intralipid
IRE1α	Inositol-requiring enzyme 1 alpha
JNK	C-Jun N-terminal kinase
KRT7	Keratin 7
KRT19	Keratin 19
LDH	Lactate dehydrogenase
LPS	Lipopolysaccharide
MAGPIX	Multiplex bead-based immunoassay
MDA	Malondialdehyde
MDH	Malate dehydrogenase
NADH	Nicotinamide adenine dinucleotide
NAFLD	Non-alcoholic fatty liver disease
NF-κB	Nuclear factor kappa B
NOB	Nobiletin
NQO1	NAD(P)H quinone dehydrogenase 1
Nrf2	Nuclear factor erythroid 2-related factor 2
PBGD	Porphobilinogen deaminase
PLP	Pyridoxal phosphate
PN	Parenteral nutrition
*PRKAA2*	5’-AMP-activated protein kinase catalytic subunit alpha-2
ROS	Reactive oxygen species
SOD1	Superoxide dismutase 1
*SREBF2*	Sterol regulatory element binding transcription factor 2
STAT3	Signal transducer and activator of transcription 3
TBP	TATA box binding protein
TGF-β1	Transforming growth factor beta 1
THLE-2	Immortalized human hepatocyte
TNFα	Tumor necrosis factor-alpha
