# Supplementary material for: Nobiletin Attenuates Inflammation and Modulates Lipid Metabolism in an In Vitro Model of Intestinal Failure-Associated Liver Disease
Source: Pharmaceutics. 2026 Jan 9;18(1):87. doi: 10.3390/pharmaceutics18010087 (PMC12844698; doi:10.3390/pharmaceutics18010087)
Supplement: Supplementary file 1 [file pharmaceutics-18-00087-s001.zip › pharmaceutics-4045942-supplementary.pdf]

pharmaceutics

Supplementary Materials: Nobiletin Attenuates Inflammation and Modulates Lipid Metabolism in an In Vitro Model of Intestinal Failure-Associated Liver Disease

Marta Belka [1,2,†], Aleksandra Gostyńska-Stawna [3,†], Karina Sommerfeld-Klatta [4,†], Maciej Stawny [3,†] and Violetta Krajka-Kuźniak [2,*,†]

1 Poznan University of Medical Sciences, Doctoral School, Bukowska 70, Poznan 60-812, Poland; mbel-ka@ump.edu.pl
2 Poznan University of Medical Sciences, Department Pharmaceutical Biochemistry, Rokietnicka 3, Poznan 60-806, Poland
3 Poznan University of Medical Sciences, Department of Pharmaceutical Chemistry, Rokietnicka 3, Poznan 60-806, Poland; agostynska@ump.edu.pl (A.G.-S.), mstawny@ump.edu.pl (M.S.)
4 Poznan University of Medical Sciences, Department of Toxicology, Rokietnicka 3, Poznan 60-806, Poland; ksommerfeld@ump.edu.pl
* Correspondence: vkrajka@ump.edu.pl
† Poznan University of Medical Sciences (https://ror.org/02zbb2597).

Table S1. Raw PCR data for gene expression analysis: (**a**) the levels of TBP,PGGD; (**b**) the levels of CYP7A1, SREPF2; (**c**) the levels of PRKAA2, ABCA1.

(a)

Number	Position	Sample Name	Gene Name	Cq	Dye	Slope	EPF
1	A1	C	TBP	33.93	SYBR Green I	0.54	2.93
2	A2	C	TBP	30.63	SYBR Green I	0.63	3.19
3	A3	C	TBP	32.3	SYBR Green I	0.67	3.28
4	A4	C	PBGD	24.68	SYBR Green I	0.66	3.28
5	A5	C	PBGD	24.72	SYBR Green I	0.89	3.85
6	A6	C	PBGD	24.69	SYBR Green I	0.94	3.97
13	B1	LPS	TBP	32.76	SYBR Green I	0.51	2.85
14	B2	LPS	TBP	35.23	SYBR Green I	0.58	3.04
15	B3	LPS	TBP	34.01	SYBR Green I	0.6	3.08
16	B4	LPS	PBGD	25.11	SYBR Green I	0.66	3.25
17	B5	LPS	PBGD	25.24	SYBR Green I	0.81	3.63
18	B6	LPS	PBGD	25.19	SYBR Green I	0.95	3.94
25	C1	INT	TBP	31.95	SYBR Green I	0.61	3.19
26	C2	INT	TBP	35	SYBR Green I	0.7	3.38
27	C3	INT	TBP	33.55	SYBR Green I	0.68	3.29
28	C4	INT	PBGD	24.5	SYBR Green I	0.79	3.54
29	C5	INT	PBGD	24.79	SYBR Green I	0.98	3.78
30	C6	INT	PBGD	24.57	SYBR Green I	0.88	3.7
37	D1	INT+LPS	TBP	31.95	SYBR Green I	0.84	3.14
38	D2	INT+LPS	TBP	33.53	SYBR Green I	0.95	3.52
39	D3	INT+LPS	TBP	32.48	SYBR Green I	0.78	3.6
40	D4	INT+LPS	PBGD	24.54	SYBR Green I	0.8	3.6
41	D5	INT+LPS	PBGD	25.05	SYBR Green I	1.04	3.78
42	D6	INT+LPS	PBGD	24.83	SYBR Green I	1.16	3.86
49	E1	INT+LPS+NOB 10µ	TBP	31.56	SYBR Green I	0.62	3.23
50	E2	INT+LPS+NOB 10µ	TBP	33.22	SYBR Green I	0.64	3.27
51	E3	INT+LPS+NOB 10µ	TBP	32.41	SYBR Green I	0.74	3.49
52	E4	INT+LPS+NOB 10µ	PBGD	24.98	SYBR Green I	0.91	3.81

53	E5	INT+LPS+NOB 10μ	PBGD	24.92	SYBR Green I	0.95	3.49
54	E6	INT+LPS+NOB 10μ	PBGD	25.1	SYBR Green I	0.91	3.9
61	F1	INT+LPS+NOB 25μ	TBP	32.2	SYBR Green I	0.62	3.19
62	F2	INT+LPS+NOB 25μ	TBP	33.15	SYBR Green I	0.62	3.18
63	F3	INT+LPS+NOB 25μ	TBP	32.69	SYBR Green I	0.81	3.43
64	F4	INT+LPS+NOB 25μ	PBGD	24.96	SYBR Green I	0.76	3.42
65	F5	INT+LPS+NOB 25μ	PBGD	25.3	SYBR Green I	0.64	3.02
66	F6	INT+LPS+NOB 25μ	PBGD	25.1	SYBR Green I	0.97	3.58

(b)

mber	Position	Sample Name	Gene Name	Cq	Dye	Slope	EPF
1	A1	C	CYP7A1	36.23	SYBR Green I	0.96	2
2	A2	C	CYP7A1	33.78	SYBR Green I	0.69	1.6
3	A3	C	CYP7A1	34.8	SYBR Green I	0.83	2.2
4	A4	C	SREPF2	22.29	SYBR Green I	0.74	2.43
5	A5	C	SREPF2	21.42	SYBR Green I	1.04	2.26
6	A6	C	SREPF2	21.88	SYBR Green I	1.14	2.03
13	B1	LPS	CYP7A1	37.83	SYBR Green I	0.05	0.11
14	B2	LPS	CYP7A1	35.21	SYBR Green I	0.41	0.71
15	B3	LPS	CYP7A1	36.21	SYBR Green I	1.01	2.08
16	B4	LPS	SREPF2	22.54	SYBR Green I	0.68	1.64
17	B5	LPS	SREPF2	21.72	SYBR Green I	0.86	2.16
18	B6	LPS	SREPF2	22.01	SYBR Green I	0.01	0.03
25	C1	INT	CYP7A1	38.33	SYBR Green I	1.38	3.78
26	C2	INT	CYP7A1	35.39	SYBR Green I	0.7	1.69
27	C3	INT	CYP7A1	36.2	SYBR Green I	0.84	3
28	C4	INT	SREPF2	22.29	SYBR Green I	0.49	0.84
29	C5	INT	SREPF2	21.19	SYBR Green I	0.01	0.02
30	C6	INT	SREPF2	21.65	SYBR Green I	0.89	2.84
37	D1	INT+LPS	CYP7A1	36.93	SYBR Green I	0.01	0.05
38	D2	INT+LPS	CYP7A1	34.19	SYBR Green I	0.01	0.04
39	D3	INT+LPS	CYP7A1	35.13	SYBR Green I	1.06	2.76
40	D4	INT+LPS	SREPF2	21.79	SYBR Green I	0	0.03
41	D5	INT+LPS	SREPF2	21.11	SYBR Green I	0.03	0.07
42	D6	INT+LPS	SREPF2	21.19	SYBR Green I	0.34	0.56
49	E1	INT+LPS+NOB 10μ	CYP7A1	36.53	SYBR Green I	1.43	1.89
50	E2	INT+LPS+NOB 10μ	CYP7A1	33.92	SYBR Green I	0.43	0.75
51	E3	INT+LPS+NOB 10μ	CYP7A1	35.11	SYBR Green I	1.32	1.92
52	E4	INT+LPS+NOB 10μ	SREPF2	22.79	SYBR Green I	0.46	0.77
53	E5	INT+LPS+NOB 10μ	SREPF2	21.72	SYBR Green I	0.98	1.49
54	E6	INT+LPS+NOB 10μ	SREPF2	22.04	SYBR Green I	0.77	1.64
61	F1	INT+LPS+NOB 25μ	CYP7A1	36.73	SYBR Green I	0.32	0.51
62	F2	INT+LPS+NOB 25μ	CYP7A1	33.79	SYBR Green I	0.66	1.95
63	F3	INT+LPS+NOB 25μ	CYP7A1	34.77	SYBR Green I	0.01	0.1
64	F4	INT+LPS+NOB 25μ	SREPF2	23.49	SYBR Green I	0.93	2.34
65	F5	INT+LPS+NOB 25μ	SREPF2	22.29	SYBR Green I	1.21	4.12
66	F6	INT+LPS+NOB 25μ	SREPF2	22.57	SYBR Green I	1	3.53

(c)

Number	Position	Sample Name	Gene Name	Cq	Dye	Slope	EPF
1	A1	C	PRKAA2	28.48	SYBR Green I	0.78	3.48
2	A2	C	PRKAA2	28.5	SYBR Green I	0.77	3.42
3	A3	C	PRKAA2	27.72	SYBR Green I	0.93	3.83
4	A4	C	ABCA1	24.57	SYBR Green I	0.82	3.32

5	A5	C	ABCA1	24.62	SYBR Green I	1.03	3.66
6	A6	C	ABCA1	24.41	SYBR Green I	1.04	3.7
13	B1	LPS	PRKAA2	29.78	SYBR Green I	1.16	4.2
14	B2	LPS	PRKAA2	29.81	SYBR Green I	0.66	3.1
15	B3	LPS	PRKAA2	28.95	SYBR Green I	0.88	3.71
16	B4	LPS	ABCA1	25.77	SYBR Green I	1.66	2.16
17	B5	LPS	ABCA1	25.69	SYBR Green I	0.91	3.37
18	B6	LPS	ABCA1	25.21	SYBR Green I	0.93	3.45
25	C1	INT	PRKAA2	30.88	SYBR Green I	1.14	4.2
26	C2	INT	PRKAA2	30.17	SYBR Green I	0.79	3.47
27	C3	INT	PRKAA2	29.1	SYBR Green I	0.92	3.8
28	C4	INT	ABCA1	25.67	SYBR Green I	1.07	3.77
29	C5	INT	ABCA1	25.51	SYBR Green I	1.08	3.76
30	C6	INT	ABCA1	25.21	SYBR Green I	0.98	3.58
37	D1	INT+LPS	PRKAA2	29.58	SYBR Green I	1.2	4.34
38	D2	INT+LPS	PRKAA2	29.32	SYBR Green I	0.8	3.52
39	D3	INT+LPS	PRKAA2	28.52	SYBR Green I	1.13	4.2
40	D4	INT+LPS	ABCA1	25.67	SYBR Green I	1.2	4.18
41	D5	INT+LPS	ABCA1	25.5	SYBR Green I	1.11	3.81
42	D6	INT+LPS	ABCA1	25.06	SYBR Green I	1.18	4.15
49	E1	INT+LPS+NOB 10μ	PRKAA2	29.78	SYBR Green I	0.76	3.39
50	E2	INT+LPS+NOB 10μ	PRKAA2	29.75	SYBR Green I	0.77	3.42
51	E3	INT+LPS+NOB 10μ	PRKAA2	28.63	SYBR Green I	0.94	3.87
52	E4	INT+LPS+NOB 10μ	ABCA1	25.37	SYBR Green I	0.84	3.24
53	E5	INT+LPS+NOB 10μ	ABCA1	25.19	SYBR Green I	1.07	3.77
54	E6	INT+LPS+NOB 10μ	ABCA1	24.91	SYBR Green I	1	3.61
61	F1	INT+LPS+NOB 25μ	PRKAA2	29.28	SYBR Green I	0.77	3.45
62	F2	INT+LPS+NOB 25μ	PRKAA2	29.21	SYBR Green I	0.65	3.07
63	F3	INT+LPS+NOB 25μ	PRKAA2	28.34	SYBR Green I	1.01	3.99
64	F4	INT+LPS+NOB 25μ	ABCA1	25.07	SYBR Green I	0.81	3.13
65	F5	INT+LPS+NOB 25μ	ABCA1	24.89	SYBR Green I	0.91	3.38
66	F6	INT+LPS+NOB 25μ	ABCA1	24.61	SYBR Green I	1.14	4.02

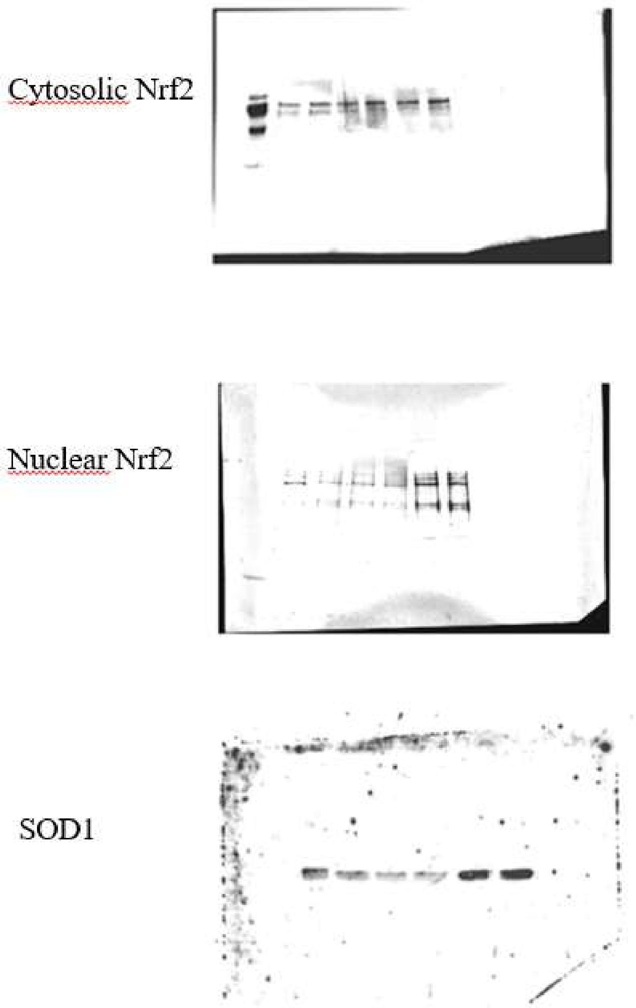

Figure S1. The representative Western blot images of cytosolic and nuclear Nrf2, and cytosolic SOD1.
